# Blood-brain barrier disruption and hemorrhagic transformation in acute stroke before endovascular reperfusion therapy

**DOI:** 10.3389/fneur.2024.1349369

**Published:** 2024-05-02

**Authors:** Yuchen Liang, Yang Yu, Jiaxin Liu, Xuewei Li, Xue Chen, Hongwei Zhou, Zhen-Ni Guo

**Affiliations:** ^1^Department of Radiology, The First Hospital of Jilin University, Changchun, China; ^2^Siemens Healthineers Ltd. CT Collaboration, Siemens Healthineers Ltd., Beijing, China; ^3^Department of Neurology, Stroke Center, The First Hospital of Jilin University, Changchun, China; ^4^Department of Neurology, Neuroscience Research Center, The First Hospital of Jilin University, Changchun, China

**Keywords:** acute ischemic stroke, blood-brain barrier disruption, perfusion computed tomography, hemorrhagic transformation, endovascular therapy

## Abstract

**Background and purpose:**

Early blood–brain barrier (BBB) disruption in patients with acute ischemic stroke (AIS) can be detected on perfusion computed tomography (PCT) images before undergoing reperfusion therapy. In this study, we aimed to determine whether early disruption of the BBB predicts intracranial hemorrhage transformation (HT) in patients with AIS undergoing endovascular therapy and further identify factors influencing BBB disruption.

**Methods:**

We retrospectively analyzed general clinical and imaging data derived from 159 consecutive patients with acute anterior circulation stroke who were admitted to the Department of Neurology of the First Hospital of Jilin University, and who underwent endovascular treatment between January 1, 2021, and March 31, 2023. We evaluated the relationship between BBB destruction and intracranial HT before endovascular reperfusion therapy and examined the risk factors for early BBB destruction.

**Results:**

A total of 159 patients with assessable BBB leakage were included. The median (interquartile range, IQR) age was 63 (54–70) years, 108 (67.9%) patients were male, and the median baseline National Institutes of Health Stroke Scale (NHISS) score was 12 (10–15). Follow-up non-contrast computed tomography (NCCT) detected HT in 63 patients. After logistic regression modeling adjustment, we found that BBB leakage in the true leakage area was slightly more than 2-fold risk of HT (odds ratio [OR], 2.01; 95% confidence interval [CI] 1.02–3.92). Heart rate was also associated with HT (OR, 1.03, 95% CI, 1.00–1.05). High Blood–brain barrier permeability (BBBP) in the true leakage area was positively correlated with infarct core volume (OR, 1.03; 95% CI, 1.01–1.05).

**Conclusion:**

Early BBB destruction before endovascular reperfusion therapy was associated with HT, whereas high BBBP correlated positively with infarct core volume.

## 1 Introduction

The incidence of ischemic stroke has increased annually along with the advancing age of the global population. The prognosis for patients with large-vessel occlusion is often poor, as severe stroke can impair consciousness, cause paralysis, and be fatal (1, 2). The prevention and treatment of stroke have recently progressed, which has led to significantly decreased morbidity and mortality rates. However, the therapeutic effects on patients with acute ischemic stroke (AIS) are still limited. Intravenous thrombolysis and endovascular therapy are currently the most effective reperfusion therapies for restoring blood flow, and endovascular therapy has a higher recanalization rate than intravenous thrombolysis (3, 4). Nevertheless, reperfusion therapy poses a risk of injury that can lead to hemorrhagic transformation (HT), resulting in neurological deterioration and increased mortality (5). Moreover, endovascular surgery increases the risk of HT (1, 2).

**Figure 1:**
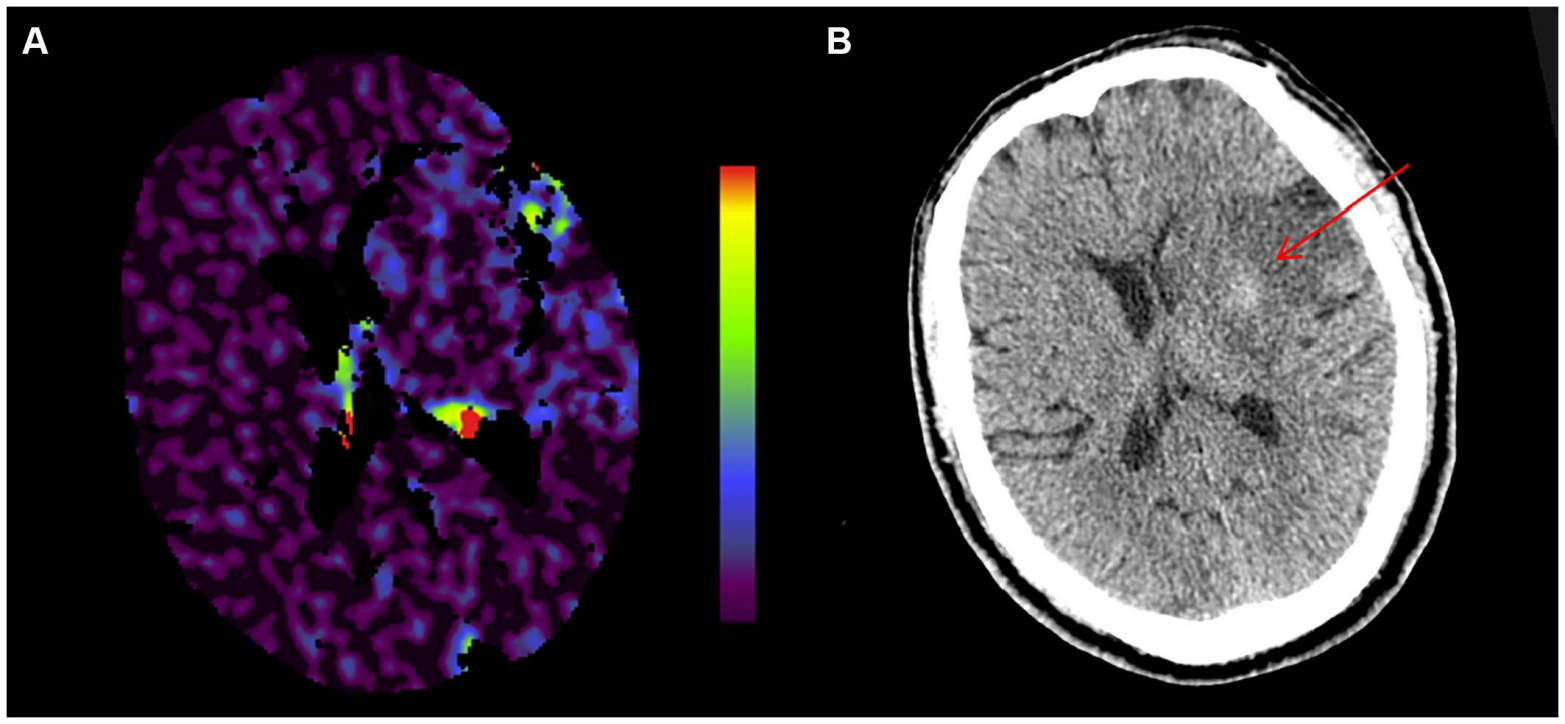
Cerebral infarction in a 74-year-old man. **(A)** Pre-endovascular treatment BBBP map. **(B)** Cranial NCCT alignment map 24 h after reperfusion therapy. A small parenchymal hemorrhage (PH1) has developed in the left basal ganglia. BBBP, blood-brain barrier permeability; NCCT, non-contrast computer tomography.

Blood-brain barrier (BBB) disruption is an important pathophysiological change during the acute phase of AIS. A dysfunctional BBB is a consequence of ischemia, but it can exacerbate parenchymal injury via peripheral immune cell infiltration that causes hemorrhage and edema. This is an important factor that influences the outcomes of therapy-induced reperfusion after stroke. The preferred test of choice for patients with acute stroke remains perfusion computed tomography (PCT), which is rapid and widely available. Furthermore, more information about BBB integrity can be obtained by extending the acquisition duration. The assessment and quantitation of cerebral perfusion using PCT *in vivo* are important in acute stroke. BBB disruption can be calculated by measuring the gradual leakage of iodinated contrast agents from cerebral vessels, and the degree of BBB disruption in patients with cerebral infarction can be assessed by generating blood-brain barrier permeability (BBBP) maps (6). The software 3D Slicer^1^ provides a convenient and reproducible post-processing method, offering the possibility of outlining regions of interest (ROIs). Destruction of the BBB is associated with both prognosis and complications of AIS (7), and further clarification of the degree of BBB disruption is important for the prognostic assessment of patients with AIS. In this study, we aimed to determine whether early disruption of the BBB predicts intracranial HT in patients with AIS undergoing endovascular therapy and further investigate factors influencing BBB disruption (see Figure 1).

## 2 Methods

### 2.1 Study population

We retrospectively reviewed patients with acute anterior circulation stroke who were admitted to Department of Neurology and received endovascular treatment at the First Hospital of Jilin University between January 2021 and April 2023. The inclusion criteria were as follows: age ≥ 18 years, AIS in the anterior circulation confirmed by diffusion-weighted imaging (DWI) sequences or CT review, treated with endovascular therapy, and complete baseline CT and CT review imaging data. The exclusion criteria comprised: contraindication to endovascular treatment, image quality insufficient for analysis, did not complete the imaging review (including stroke in which distinguishing between cerebral hemorrhage and contrast extravasation was difficult without follow-up imaging information), and bilateral or posterior circulation ischemic stroke. We also included general clinical information and PCT imaging data on admission and non-contrast computed tomography (NCCT) within 72 h of follow-up. The study was approved by the local ethics committee and informed consent was obtained.

### 2.2 Imaging protocol

Patients were assessed using standardized imaging protocols for NCCT upon admission, computed tomography angiography (CTA), PCT, and follow-up NCCT within 72 h. Patients with acute stroke underwent endovascular treatment according to current stroke guidelines. The CT, CTA, and PCT images were acquired using a SOMATOM Force Dual Source CT scanner (Siemens Healthineers AG., Forchheim, Germany).

Three seconds after the start of the injection, sequential PCT imaging proceeded using the following parameters: 70 kVp, 200 mAs, 1.5-s scans, rotation time 0.25 s, and layer thickness 3 mm. The PCT images were post-processed using syngo.via software (Siemens) and a deconvolution modeling algorithm.

### 2.3 Evaluation of BBB disruption

Image post-processing using a syngo.via CT Neuro Perfusion deconvolution model determined cerebral blood flow (CBF), cerebral blood volume (CBV), mean time to passage (MTT), time to maximum of the residue function (Tmax), and generated BBBP map. The severe hypoperfusion zone was generated using Tmax >6 s and an ischemic core using the absolute value of CBV < 2 mL·100 g^−1^, and regarded the severe hypoperfusion zone minus the infarct core as the penumbra (7). The BBBP measurements were extracted from the PCT data based on a deconvolution model that uses arterial and tissue time attenuation curves to calculate the outflow rate of the contrast agent from the intravascular to the extravascular space, which is used as an indicator of the BBBP value. Values for BBBP are expressed as mL·100 mL^−1^·min^−1^. Our assessment of BBB consists of two parts: ① The penumbra and infarct core regions were used as ROIs and superimposed on the permeability map, which in turn calculated the extent of BBB disruption within the ROIs (8). ② True leakage area assessment: we applied a new ROIs evaluation method to further analyze the BBB disruption. The PCT perfusion data were imported into 3D Slicer software (mainly Tmax, CBV and BBBP maps), and the penumbra and infarct core were obtained by the same calculation method. Because BBBP is a measure of BBB integrity, and BBBP in the brain is normally almost zero (9, 10), we attempted to filter out the region with BBBP of 0 mL·100 mL^−1^·min^−1^ in the penumbra by setting a threshold value using 3D Slicer, which reflects true BBB destruction in the penumbra. This part of the range we call the true leakage area, and use this to align to the BBBP to further analyze the BBB destruction in the true leakage area. Our primary outcome was the occurrence of HT on NCCT images within 72 h. According to the European Cooperative Acute Stroke Study (ECASSII) criteria, the severity of HT on CT images was classified into two stages: hemorrhagic infarction (HI) and parenchymal hemorrhage (PH) with or without mass effects (11). Each stage was divided into two subtypes. Images were consistently scored by three neurologists who were unaware of the clinical treatment and outcome data. If they could not agree about the results of the HT score assessment at 72 h (e.g., difficulty in distinguishing contrast extravasation from HT), they checked whether the patient had further imaging to reach a consensus.

### 2.4 Statistical analysis

Study subjects were categorized into groups with and without HT, and with and without PH-2 according to whether they developed HT or PH-2, and BBBP was categorized into high and low groups according to the mean value. Variables were compared between groups using independent sample t-tests and continuous variables with a normal distribution are expressed using Χ ± *s*. Continuous variables without normal distribution were compared between groups using Mann–Whitney U-tests and are expressed as medians with IQRs. Comparisons of categorical variables between groups were performed using the chi-square test or Fisher’s exact test and expressed as frequencies (%). Variables satisfying *p* < 0.1 in univariate analyses were included in multivariate analyses, with the presence or absence of HT, PH-2, and the degree of BBB destruction considered as dependent variables and statistically analyzed using stepwise regression in binary multivariate logistic regression, with statistically significant results expressed as odds ratio (OR) and 95% confidence interval (CI). All data were statistically analyzed using SPSS 24.0 (IBM Corp., Armonk. NY, United States), and *p* < 0.05 was considered statistically significant.

## 3 Results

Among 211 initially enrolled patients with anterior circulation stroke who had undergone endovascular treatment, 52 met the exclusion criteria, 29 had bilateral strokes, and 23 with poor images were not followed-up or did not meet the inclusion criteria, resulting in a final analysis of 159 patients. The characteristics of the study population are shown in Table 1. The median (IQR) age was 63 (54–70) years, 108 (67.9%) were male, and the median (IQR) baseline National Institutes of Health Stroke Scale (NHISS) score was 12 (10–15). Follow-up NCCT images revealed associated HT in 63 (39.6%) patients, among whom 6, 21, 12, and 24 had HI-1, HI-2, PH-1, and PH-2, respectively. The mean (± SD) penumbra volume was 89.0 ± 51.9 mL, and patients with and without HT did not significantly differ (84.3 vs. 92.2 mL, *p* = 0.350). The mean (± SD) core volume was 26.8 ± 21.7 mL, and patients with and without HT did not significantly differ (29.3 vs. 25.2 mL, *p* = 0.237). The mean (± SD) penumbra BBBP was 1.3 ± 0.5 mL·100 mL^−1^·min^−1^, and the mean (± SD) core BBBP was 1.3 ± 0.6 mL·100 mL^−1^·min^−1^. Patients who developed HT had greater destruction of the BBB in the penumbra region (1.4 vs. 1.2 mL·100 mL^−1^·min^−1^, *p* = 0.068) and higher BBBP in the infarct core (1.3 vs. 1.2 mL·100 mL^−1^·min^−1^, *p* = 0.324) compared with those who did not develop HT, but there was no significant difference between the two groups. The mean (± SD) true leakage area BBBP was 1.8 ± 0.5 mL·100 mL^−1^·min^−1^, with a significant difference visible between the presence and absence of HT (1.9 vs. 1.7 mL·100 mL^−1^·min^−1^, *p* = 0.019). The median (IQR) heart rate was 79 (70–87) min^−1^ with a statistically significant difference between the presence and absence of HT (80 vs. 78 min^−1^, *p* = 0.008). Table 2 shows the results of the multivariate findings of HT. After adjusting for confounders, true leakage area BBB disruption was associated with a 2-fold greater risk of HT (OR, 2.01; 95% CI, 1.02–3.92, *p* = 0.041). Heart rate was also associated with HT (OR, 1.03; 95% CI, 1.00–1.05, *p* = 0.033). In addition, PH-2, the most severe subtype of HT, is closely related to worse outcome (6). In our study (Table 1), although the degree of destruction of the BBB in the true leakage area was greater in patients who developed PH-2 compared with those who did not (2.0 vs.1.7 mL·100 mL^−1^·min^−1^, *p* = 0.054), it was not statistically significant. There were significant differences in coronary heart disease and platelet distribution width between the PH-2 group and the no PH-2 group (all *p* < 0.05). However, after correcting for confounders, we did not find a correlation between clinical and imaging indicators and the PH-2 group (Table 3).

**Table 1:** General and imaging characteristics of the patients.

	HT				PH-2			BBB disruption		
Variables	Total	No HT	HT	p	No PH-2	PH-2	P	Low BBBP	High BBBP	p
Age, y	63 (54,70)	62(53,70)	65(55,70)	0.409	63 (53,70)	65 (57,69)	0.433	63 (53,70)	67 (55,75)	0.797
Men, *n* (%)	108 (67.9)	63 (65.6)	45 (71.4)	0.443	93 (68.9)	15 (62.5)	0.537	54 (60.0)	54 (78.3)	**0.014**
HR, min^−1^	79 (70,87)	78 (68,86)	80 (72,96)	**0.008**	79 (70,87)	79 (70,99)	0.460	80 (72,87)	80 (71,87)	0.865
FBG, mmol/L	6.5 (5.6, 9.0)	6.4 (5.4, 8.1)	7.4(5.4,8.1)	**0.046**	6.5 (5.5,8.8)	7.1 (5.6,9.3)	0.454	6.6 (5.6,8.6)	7.1 (5.8,9.3)	0.247
CHD, *n* (%)	20 (12.6)	11 (11.5)	9 (14.3)	0.599	14 (10.4)	6 (25.0)	**0.047**	9 (10.0)	11 (15.9)	0.263
Smoker, *n* (%)	79 (49.7)	49 (51.0)	30 (47.6)	0.673	69 (51.1)	10 (41.7)	0.394	43 (47.8)	36 (52.2)	0.583
Hypertension, *n* (%)	96(60.4)	60(62.5)	36(57.1)	0.499	84 (62.2)	12(50.0)	0.259	57 (63.3)	39 (56.5)	0.384
Hyperglycemia, *n* (%)	30 (18.9)	20 (20.8)	10 (15.9)	0.434	26 (19.3)	4 (16.7)	0.766	18 (20.0)	12 (17.4)	0.677
Anticoagulation, *n* (%)	50 (31.4)	25 (26.0)	25 (39.7)	0.070	45 (33.3)	5 (20.8)	0.246	24 (26.7)	26 (37.7)	0.138
Platelets, ×10^−9^/L	211.3 ± 50.4	215.7 ± 54.0	204.3 ± 43.8	0.103	214.2 ± 50.9	196.0 ± 49.1	0.105	218 ± 52.5	200 ± 46.3	**0.013**
PDW, (%)	11.7 ± 1.6	11.5 ± 1.4	12.0 ± 1.9	**0.011**	11.6 ± 1.6	12.3 ± 2.2	**0.047**	11.5 ± 1.8	12.0 ± 1.5	**0.046**
NEUT, ×10^−9^/L	7.0 ± 2.9	6.6 ± 2.8	7.7 ± 3.0	**0.028**	7.0 ± 3.0	6.8 ± 2.7	0.708	7.0 ± 2.9	7.1 ± 2.9	0.710
Baseline NIHSS	12 (10,15)	12 (9,15)	13 (11,16)	**0.034**	12 (10,15)	13 (12,16)	0.250	12 (9,15)	13 (11,16)	**0.049**
Penumbra*	1.3 ± 0.5	1.2 ± 0.5	1.4 ± 0.5	0.068	1.3 ± 0.5	1.5 ± 0.5	0.092	–	–	–
Infarct core*	1.3 ± 0.6	1.2 ± 0.6	1.3 ± 0.5	0.324	1.2 ± 0.6	1.4 ± 0.6	0.167	–	–	–
True leakage area*	1.8 ± 0.5	1.7 ± 0.5	1.9 ± 0.5	**0.019**	1.7 ± 0.5	2.0 ± 0.6	0.054	–	–	–
Penumbra volume, mL	89.0 ± 51.9	92.2 ± 55.6	84.3 ± 45.8	0.350	88.8 ± 53.6	90.2 ± 42.7	0.907	78.3 ± 47.5	101.5 ± 55.3	**0.020**
Infarct core volume, mL	26.8 ± 21.7	25.2 ± 19.9	29.3 ± 24.1	0.237	26.0 ± 21.0	31.7 ± 25.3	0.907	20.6 ± 19.8	35.0 ± 21.2	**<0.001**
Hypoperfusion area, mL	115.9 ± 62.7	117.4 ± 64.6	113.6 ± 60.1	0.715	114.8 ± 64.6	121.9 ± 51.3	0.609	98.9 ± 55.6	136.5 ± 64.1	**<0.001**

*Data are shown as mL·100 mL^−1^·min^−1^. All other data are shown as medians with interquartile ranges, means ± standard deviation, or as numbers (%). Bold type, significant at (*p* < 0.05). BBB, blood brain barrier; BBBP, blood-brain barrier permeability; CHD, coronary heart disease; FBG, fasting blood glucose; HR, heart rate; HT, hemorrhage transformation; NEUT, neutrophil; NIHSS, National Institutes of Health Stroke Scale; PDW, platelet distribution width; NS, not significant.

**Table 2:** Multivariate risk analysis of HT.

	Dependent variable: HT		
Variables	OR	95% CI	p
HR	1.03	1.00–1.05	**0.033**
PDW	1.21	0.98–1.50	0.080
NEUT	1.12	1.00–1.26	0.064
True leakage area	2.01	1.02–3.92	**0.041**

Bold indicates significant value (*p* < 0.05). HT, hemorrhage transformation; OR, odds ratio; CI, confidence interval; HR, heart rate; PDW, platelet distribution width; NEUT, neutrophil.

**Table 3:** Multivariate risk analysis of PH2.

	Dependent variable: PH2		
Variables	OR	95% CI	p
CHD	2.70	0.91–8.03	0.074
True leakage area	2.16	0.93–5.02	0.074

OR, odds ratio; CI, confidence interval; CHD, coronary heart disease.

Our ultimate goal in studying BBB disruption was to reduce the risk of HT and improve the prognosis of patients with acute stroke. Imaging metrics offer the possibility of quantifying BBB disruption, and combining them with clinical laboratory values to identify factors that can help to protect the integrity of the BBB is a promising therapeutic goal. We did not find a correlation between BBB destruction and HT in the penumbra region. Therefore, we categorized subgroups of BBB destruction in the true leakage area. To further investigate factors that influence BBB disruption, we categorized the subgroups according to mean values indicating low and high disruption (BBBP <1.769 and > 1.769 mL·100 mL^−1^·min^−1^, respectively) and explored correlations between clinical and imaging indexes (Table 1). Men, platelet count, platelet distribution width, penumbra volume, infarct volume, total hypoperfused area, and baseline NHISS scores upon admission significantly differed between the two BBB regions (all *p* < 0.05). After adjusting for confounders (Table 4), High BBBP in the true leakage area was associated with infarct core volume (OR, 1.03; 95% CI, 1.01–1.05, *p* = 0.001).

**Table 4:** Multivariate risk analysis of BBB destruction.

	Dependent variable: BBB destruction		
Variables	OR	95% CI	p
Men	0.53	0.25–1.12	0.096
Platelets	0.99	0.98–1.00	0.061
Infarct core volume	1.03	1.01–1.05	**0.001**

BBB destruction as a dichotomous variable (Low vs. High). Bold indicates significant value (*p* < 0.05). OR, odds ratio; CI, confidence interval.

## 4 Discussion

This retrospective observational study of patients with AIS undergoing endovascular reperfusion therapy confirmed previous findings of an association between BBB disruption and HT in patients with acute stroke (12), which increases the risk of developing HT by more than twofold. The ischemic penumbra is the transition zone between the ischemic core and normal tissue, and unlike the rapid cell death of the ischemic core, the ischemic penumbra can last for days or even months and can be salvaged. Modern stroke research often bases clinical treatment strategies on this area, which has become the focus of imaging evaluations (13). Unlike previous studies, we defined the true leakage area by filtering out regions with zero BBB values in the penumbra and generated higher BBB values than before. A previous study manually outlined and averaged three circular ROIs of 10 mm in diameter by mapping a severely hypoperfused region of the BBB. However, we believe that the reproducibility of that method is challenged because not all patients experience more severe BBB disruption at these three levels, some patients have more severe BBB disruption, and these three levels do not reflect the severity of overall BBB disruption. Our approach is an extension of that study (7). We therefore used a new method to select ROIs by filtering the penumbra region based on a BBB value of 0 mL·100 mL^−1^·min^−1^. The true leakage area data were obtained using 3D Slicer, which guarantees reproducibility and a closer approximation of destruction of the entire penumbra region BBB. We also found a non-significant association between ischemic core BBB disruption and HT, which was consistent with previous findings and contrary to earlier results (7, 14). This is possibly because the infarct core region tends to be accompanied by more severely disrupted blood flow, which is more difficult to determine by short scans with contrast medium. Clinicians must weigh the potential benefits and risks of reperfusion therapy, which also emphasizes the importance of BBB assessment. Assessing BBBP changes in the ischemic zone in patients with acute stroke helps to predict those who are more likely to develop HT after reperfusion therapy. We also realized that faster heart rate was valuable for predicting HT. Abnormal heart rate reflects not only changes in cardiac function but also altered autonomic dysfunction due to abnormal brain function. Previous studies have shown that heart rate in the acute phase is a predictor of major clinical events in patients with ischemic stroke (15–17), but relatively few studies have been conducted on the relationship between heart rate and HT in patients with AIS. The ambulatory heart rate during the acute phase can be conveniently monitored, and can help to make an early and timely judgment regarding the patient’s prognosis, thus guiding clinical management strategies in real time. In addition, PH-2, the most severe form of HT, is associated with worse outcomes (6). After univariate analysis, we observed a significant association between coronary heart disease and platelet distribution width between the presence and absence of PH-2. However, upon adjusting for potential confounders, no statistically significant findings were obtained. The limited sample size and imbalance may have contributed to these results. In future investigations, it is imperative to expand the sample size to enhance the validity of our findings.

We found a correlation between HT and BBB disruption, and ultimate goal of clinical studies of BBB disruption is to further improve patient prognosis by mitigating it. We further investigated factors affecting the BBB and categorized true leakage area BBBP values as lower or higher. Infarct volume was positively correlated with the degree of BBB disruption after adjustment for confounders. A larger infarct area tends to imply worse blood flow disruption, and ischemic injury leads to more severe BBB disruption through a cascade of cellular and metabolic disturbances such as the production of proteases, free radicals, and various inflammatory factors that disrupt the integrity of the basement membrane and tight junctions (18). Since BBB disruption is associated with hemorrhage after reperfusion therapy, reducing the risk of HT in patients by protecting against BBB disruption might be a future therapeutic direction. Therefore, we investigated factors affecting BBB disruption in anticipation of facilitating clinical treatment strategies.

This study has several limitations. The retrospective design included selectivity bias, and the small study cohort was limited to patients with acute stroke undergoing endovascular therapy. Thus the patient population needs to be larger and should include patients with stroke who underwent different types of therapy to provide a comprehensive evaluation of stroke patients undergoing reperfusion. In addition, some uncertainty remains in the assessment of contrast extravasation versus HT, and we have tried to minimize this error.

Contrast agent extravasation is a surrogate indicator of BBB disruption, and extravasated tracer results in enhanced image contrast. PCT further calculates BBBP by quantifying the rate of contrast agent efflux from plasma to tissue. Although MRI might provide a more accurate estimate of BBB disruption, it requires longer imaging acquisition than the faster PCT protocols. Therefore, PCT is currently an accurate and convenient method for analyzing the status of patients with time-dependent acute stroke (19).

Our findings confirmed a correlation between BBB destruction and subsequent HT in patients with AIS undergoing reperfusion therapy. The main clinical implication of this finding is that patients who are more likely to be at risk after reperfusion therapy should be identified. In addition, we investigated factors that influence BBB destruction and anticipate that these factors will facilitate the clinical management of future patients with stroke.

## Data availability statement

The raw data supporting the conclusions of this article will be made available by the authors, without undue reservation.

## Ethics statement

The studies involving humans were approved by Ethics Committee of the First Hospital of Jilin University The First Hospital of Jilin University. The studies were conducted in accordance with the local legislation and institutional requirements. The participants provided their written informed consent to participate in this study.

## Author contributions

YL: Writing – original draft. XL: Writing – review & editing. HZ: Writing – review & editing. Z-NG: Writing – review & editing. YY: Methodology, Formal analysis, Writing – review & editing. JL: Methodology, Formal analysis, Writing – review & editing. XC: Data curation, Writing – review & editing.

## Footnotes

^1^https://slicer.org
